# Mission‐oriented translational cancer research – health economics

**DOI:** 10.1002/1878-0261.12440

**Published:** 2019-02-20

**Authors:** Bengt Jönsson, Richard Sullivan

**Affiliations:** ^1^ Department of Economics Stockholm School of Economics Sweden; ^2^ Institute of Cancer Policy King's College London UK

**Keywords:** cancer policy, cancer research, health economics, translational cancer research

## Abstract

Health economics is an integrated aspect of all phases of mission‐oriented translational cancer research and should be considered an intrinsic component of any study aimed at improving outcomes for patients and intervention costs. Information about value and value for money of new options for prevention and treatment is needed for decisions about their adoption and use by healthcare systems.

**Abbreviations**
CDF: cancer drugs fund
CVD: cardiovascular disease
DALY: disability‐adjusted life‐year
HTA: health technology assessment
ICHOM: International Consortium for Health Outcomes Measurement
IMI: Innovative Medicines Initiative
OS: overall survival
QALY: quality‐adjusted life‐year

## 1 Introduction

Investments in cancer research are undertaken in an international context and financed by both public and private, non‐profit and for‐profit, organisations. During the last decade, the fast growth in investments in the for‐profit life science industries, particularly the pharmaceutical industry, has outpaced the slow growth in public‐funded cancer research in most countries. The growing number of new medicines and other technologies (surgical, radiotherapy) developed, together with demands for rapid introduction and use, makes decisions between the fast‐increasing number of alternatives to spend healthcare resources one of the key issues in health policy. The development of target therapies and personalised medical care, and high costs per treated patient add to the challenges.

Assessing cost‐effectiveness based on information from clinical research studies is a challenge, but much more needs and can be done by considering health economic aspects in the development of new options for prevention and treatment. It is too late to think about the need for information for decisions about adoption and use when a new medicine is ready for use in clinical practice. The choice of which studies to perform, the design of the clinical studies, and the choice of outcome measures must be made with information needs for healthcare systems – value and affordability – in mind. Otherwise there will be a growing gap in the translational research process.

It is an unavoidable fact of the development of cancer research that there will be a growing uncertainty around long‐term outcomes and clinically meaningful benefit with the new generation of complex interventions for cancer. There is thus a need to undertake follow‐up studies in clinical practice to find out the true value for patients and healthcare systems in the real world. Such studies are part of the extended translational research process and will also give information to the healthcare systems on how to optimise the use of an increasing number of options for prevention and treatment of cancer. Healthcare systems aimed at improving outcomes and value for money need information to ensure that resources are spent in an efficient and equitable way.

The European Union has a common market for medical products, services and healthcare human resources, but healthcare systems are national, driven by local politics, with different availability of resources and organisation and financing of health care. However, countries share the same knowledge about options for cancer management and thus have incentives to share information about how these options should be used for improvement of outcomes. Information from a mission‐oriented translational research project can thus benefit all countries regardless of availability of resources. But there are challenges that must be addressed, for example, the slow adoption and limited access to new cancer medicines in countries with lower levels of income and healthcare spending. Health economic issues related to prices, equal access and an efficient use of new options for cancer prevention and management are important issues in the translational research process.

## 2 Cancer research – an investment in health

From a health economics perspective, mission‐oriented translational cancer research is an investment undertaken to produce (future) improvements in health outcomes for cancer patients. Typical for investments are that the resource inputs, the costs, come before the intended consequences. Due to the time frame, there is always uncertainty about future consequences.

Investments aimed at improvements in health have several characteristics that make it difficult to predict the health outcomes. Medical research at the basic level aims at improvements in knowledge which only indirectly can be related to specific health benefits. For more applied research projects, such as clinical studies of efficacy and safety of a new medicine, it may be possible to make predictions, but only with great uncertainty around the estimates. Even when a new cancer medicine comes to the market, it may take many years before the real health outcomes can be observed. Clinical studies and trials are only an (often poor) approximation of the real world. Furthermore, such studies are often initiated and financed by public funds, as was the case with studies of long‐term effects of tamoxifen (Davies *et al*., 2013).

Investments in health are similar to other investments in that they require input of resources of different kinds, which need to be paid back while research is being carried out. Investments aimed at future production of goods and services are often financed through borrowing on different financial markets. The loans are paid back when income is generated from sale of products and services. Investments in research and development, particularly early research, are financed through government expenditure and private donations, whereas later research projects are financed through investments in the life science industry. These private and public funders of research cooperate and compete in an international setting, making it difficult to get clear picture of what resources are used for cancer research. It is even more difficult to determine the contributions of different research projects and investments to the final value of activities for prevention and treatment undertaken in the healthcare sector.

Table 1 shows an estimate of private and public funding of cancer research in the EU in 2015. The figures are only indicative, as there has not been an assessment since 2005 (Eckhouse *et al*., 2008). But the trend is clear. Investment in cancer research by the life science industry, in particular the pharmaceutical industry, has increased very quickly, whereas private not‐for‐profit and public cancer research has only increased at a slow pace. As an example, in 1997, industry funding in USA accounted for 31% of all cancer research funding, compared with 41% for National Cancer Institute (NCI) (McGeary and Burstein, 1999). In 2015, NCI spent USD 6 billion, whereas the US pharmaceutical industry spent 12 billion, assuming that 25% of spending was on cancer. Some industry executives indicate that cancer accounts for up to 40% of total research spending. This spending has resulted in a global market for cancer medicines, which in 2015 was USD107 billion. While the increase in private for‐profit spending for cancer research is of great importance for the creation of valuable medicines for patients, questions about the efficiency, cost‐effectiveness and long‐term sustainability of this change in cancer research funding have been increasing. All investments in cancer research must be motivated by their impact on health outcomes for cancer patients.

**Table 1:** Funding for cancer research in EU 2005 and 2015 (or other available years). Million Euro

Source of funding	2005	2015
Public	1000	1500
Private non‐profit	900	1800
Private for profit	2200 (10% of total)	
		8500 (25%)
		13 500 (40%)
Total	4100	11500–16 800

## 3 Resource allocation in research – a mission‐oriented approach

Researchers and research funders alike know that resources for investments in research are limited. Careful evaluation of different project proposals is necessary, and in the end only a few projects can be supported. Research managers in the life science industries have the same experience. Only a selected number of projects can be supported, and as science develops and the cost of research increases, choice become more difficult, despite the increase in spending on cancer research. As choices are an unavoidable fact of life and in cancer research as well, the question is not whether we need to make choices, but whether we can make better choices if they are informed by systematic information about the cost and outcome of different alternatives for resource allocation as well as other upstream factors such as portfolio balance and unmet research needs.

The essence of a mission‐oriented approach to translational cancer research is to evaluate different options in terms of their costs and their potential impact on the stated mission. Such evaluations are difficult at early stages of research, but even if precise predictions are difficult, thinking early on about the potential contribution to the mission is important. In an evaluation of the Innovative Medicines Initiative (IMI), this was one of the main conclusions, based also on demands from different stakeholders about evidence of what the different projects had contributed to patients and healthcare payers (IMI 2016).

When the translational research process comes into the later stages, before introduction of a new treatment to the healthcare system, such evaluations become both more feasible, as clinical studies must be designed with defined indications and alternatives in mind. Information about the likely outcome and value for different patients is also requested by healthcare providers for their decisions to pay for and adopt the new option. Also, healthcare systems face limited resources, and expectations for improved outcomes mean it necessary to make decisions about allocation of resources based on outcome and value in relation to costs. In the era of universal health coverage, countries have to balance a variety of elements in this framework; equity, quality, supply security, sustaining innovation and research, maximising access and rational use of technologies. New options for prevention and treatment based on investments in research and development must thus be evaluated in terms of available options. Relevant information for these choices comes from studies and results generated in the research process.

A mission‐oriented approach for translational cancer research is thus in line with the shift towards value‐based health care. The ideal situation would be if the introduction of a new treatment option in clinical practice was combined with all information needed for decisions about pricing and reimbursement (payment), which are the instruments used for management of the introduction of new medical technologies. However, as evidential requirements for regulatory authorisation for medicines in general, and for cancer medicines in particular, decrease, such complete *a priori* knowledge is declining. Even early in the development process, if there is information that a new treatment option may work for patients with no other therapeutic options, this may still present the best chance for improved survival and/or quality of life. Increasingly, it is also extremely costly and impractical to undertake all the studies needed to get the information. Decisions must be made on incomplete data and that makes it necessary to undertake follow‐up studies in real‐world, clinical practice. The healthcare system, as payer and provider, is thus an increasingly important partner in translational cancer research. The translational research process is not completed until evidence about impact on relevant outcomes in clinical is available (Ringborg, 2019). In addition, it is not until the clinical practice phase that other factors come into play that impact value and affordability, namely, how a country [post health technology assessment (HTA)/prioritisation] approaches its price negotiation model(s), e.g. market competition, budget capping, dose capping and managed entry agreements (value‐based purchasing etc.).

There is a gap in the translational cancer research process in which information from health economics can help to facilitate decisions, between completion of the clinical research phase and before introduction to clinical practice. Relevant information for decisions about outcome, value and cost‐effectiveness must be available. Otherwise this gap may become a bottleneck in the translational research process. A second gap where health economics can help is in providing relevant information about outcomes in clinical practice. These outcomes are not only determined by the knowledge embodied in the new medicine through scientific understanding of mechanisms of actions and results from clinical trials, but also from how the medicine is used in clinical practice. For example, if the medicine is used for the wrong patients or in the wrong way (timing, doses, etc.) or if management of patients is inadequate, for example leading to inadequate persistence and compliance, the expected outcomes may not be achieved. There are also cases where experience of use in clinical practice may produce better outcomes and/or lower costs than predicted from clinical studies.

The relevant perspective in economic evaluation is the one which can identify consequences of the intervention, prevention or treatment. For cancer treatment this means, in most cases, from diagnosis to death. Long‐term consequences should thus be included in the analysis, and estimates of cost per life year or quality‐adjusted life‐year (QALY) gained from a new programme needs models or follow up that cover the whole period to end of life. Issues related to survivorship, such as reductions in quality of life, limitations in ability to work or costs for treatments and rehabilitation related to the disease should be included. Economic aspects of cancer survivorship should thus be included in the long‐term consequences.

Health economics can thus be seen as an integrated aspect of all phases of translational research and should be considered an intrinsic component of any study which is conducted to improve outcomes for patients and costs of the intervention. The earlier in the research process questions about value and costs are addressed, the greater the possibility for a smooth development in the translational research process. This may save resources through elimination of studies that are not likely to give relevant data, e.g. poor design, and by augmenting studies with additional relevant variables so that healthcare decision makers can make informed decisions about the introduction and use in the healthcare system. Studies of impact in clinical practice should ideally be planned early in the research process and build on a coherent research plan leading to the fulfilment of the mission.

## 4 Mission and metrics – a health economics perspective

The most important outcomes are reductions in mortality due to cancer and increases in survival for cancer patients, and/or improvements in health‐related quality of life during and after treatments that are affordable for patients and healthcare systems, and equitable across the socio‐economic spectrum.

It is important to make a distinction between the measurement of the impact of these changes on health outcomes and the subjective value of the changes for individuals and society. For a child cured of cancer, it is the opportunity to live a full life, and the happiness for family and friends gained from sharing this. For a person of working age, it is not only the value of increased survival benefit but also the value of being able to work during and after treatment. For an older person, it is the value of adding years to life and life to years.

The choice of outcome measures for a mission‐oriented approach for cancer research will determine the direction of the research undertaken, as well as the value for individuals and society from progress according to the defined objective(s). If the objective is to reduce incidence and mortality due to cancer, it may be particularly relevant to focus on research that can produce new knowledge for development of preventive actions and public health, inside or outside the healthcare system.

If the mission/objective is to improve survival, focus may be on improvement of treatments, models and/or pathways of care where there are specific opportunities for increased survival. They may be different for different patients with cancer, for example young and older. How should we look at improvements in survival for patients with different survival probabilities, for example breast versus lung cancer? Should we put a specific emphasis on options to cure patients? If we are only focusing on survival, impact on quality of life will be ignored. If we include health‐related quality of life, we need to define the relative value of increase in life expectancy versus quality of life. These are no abstract academic discussions. There is substantial evidence that outcome measures utilised now are less about delivering clinically meaningful benefit and more about ‘keeping therapies’ in the research pipeline; the so‐called paradigm of ‘promissory science’. The nuances and complexity of these arguments are profound and depend on stage of disease (curative versus palliative), site‐specific cancer, socio‐demographic profiles, systems affordability, etc.

**Figure 1:**
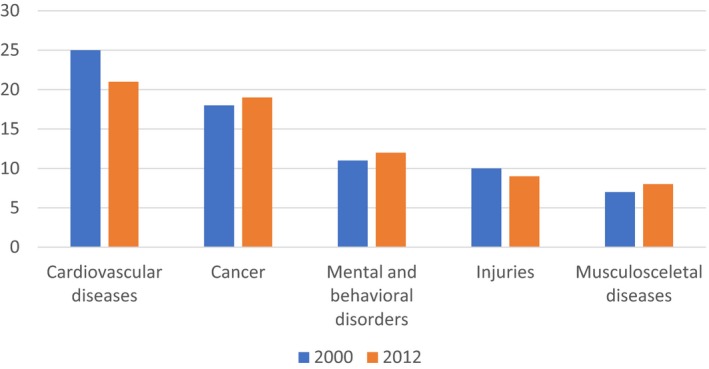
Disease burden of the top five disease groups in Europe 2000 and 2012. Source: World Health Organization (2014).

The World Health Organization defines the burden of disease as disability‐adjusted life‐year (DALY) lost due to different diseases. This metric has the advantage of including both mortality and morbidity. Both the methodology and data for calculations of DALY have shortcomings as a measure of the health burden of cancer, but estimates are done in a consistent manner using available epidemiological data on mortality and morbidity. They can thus serve as a starting point for defining and relevant metric for a mission‐oriented approach to translational cancer research. Figure 1 below shows the changing disease burden in Europe between 2000 and 2012, measured as number of DALY lost. The share of cardiovascular disease (CVD) has declined, while cancer has increased, and in some countries, for example the Netherlands, cancer has surpassed CVD and accounts for the largest share.

Looking in more detail into estimates of the DALY lost due to cancer reveals that mortality accounts for 97% of the DALY lost due to cancer, and morbidity for only 3%. The implication here is that measures such as mortality and survival rates should correlate well if the purpose is to improve outcome over time. These measures can be calculated in different ways, with different resulting metric and different interpretations. For example, age‐adjusted mortality may better represent the impact of prevention and treatment, but crude rates measure the actual number of patients the healthcare system needs to care for. The conclusion is that from an economic perspective, a wide range of metrics are needed but, most crucially, these metrics of outcomes should be well validated and should as close as possible reflect reality.

## 5 Effectiveness and efficiency – what is a relevant outcome for health economics?

It is not up to the economist or oncologist to decide which outcome measure should be used for informing decisions about allocation resources for cancer research and cancer care. This is a political decision for payers and societies, i.e. the population that finances the resources used and also is the recipient of improvements in health. However, oncologists have important knowledge of importance for defining relevant outcome measures. Traditionally, oncologists, together with clinical epidemiologists and biostatisticians, make decisions about measures of outcome or effectiveness used in clinical studies designed to evaluate efficacy and safety of methods and medicines. Lately, we have seen a new wave of outcome measurement aimed at defining patient‐relevant outcomes for use in healthcare management in a shift towards value‐based health care. Although patient‐reported outcome measures are not new – essentially these are, or should be, captured by properly powered trials with genuine equipoise and outcomes (length of life and/or quality of life) that are meaningful – the tilt to better capture real world patient measures better reflects the real world balance between length of life and cumulative toxicity/tolerability (e.g. impact of multiple grade 1 and 2 toxicities) that is overlooked by biostatistically driven clinical trial outcome measures.

Value was defined as outcome divided by cost in an often‐cited paper on value‐based care (Porter, 2010). Although this definition may be enough when using the concepts for management consultancy, it is insufficient as a guidance for health economics of translational research. This is illustrated by lessons from the National Health Service Cancer Drugs Fund (Aggarwal *et al*., 2017). Only 18 (38%) of the 47 indications approved by the Cancer Drug Fund (CDF) reported a statistically significant overall survival (OS) benefit, with an overall median survival of 3.1 months. The National Institute for Health and Care Excellence had previously rejected 26 (55%) of the CDF‐approved indications because they did not meet cost‐effectiveness thresholds. Four drugs – bevacizumab, cetuximab, everolimus and lapatinib – were approved for a total of 18 separate indications, of which 13 were subsequently deleted due to insufficient evidence of clinical benefit.

Outcome definition and measurement does not transform healthcare systems. Different outcomes have different values, and policy and management decisions should be based on results from studies providing evidence about impact of potential actions and allocation of resources. For a review of issues related to value in cancer from a health economic perspective, see Jönsson (2017). Outcome measures for several diseases have been defined for many diseases, including cancers ICHOM (2017). Typically, outcome measures for value‐based health care include measures for many different dimensions affected by the disease and its treatment.

The International Consortium for Health Outcomes Measurement (ICHOM) standard set is a recommendation of the outcomes that matter most to patients with lung cancer. Measuring these outcomes to better understand how to improve the lives of their patients is a reasonable recommendation to providers. However, measurement is costly, primarily in time for providers, and interpretation of results far from straightforward, as outcomes may move in different directions. For policy decisions, one major problem is the lack of comparability between different diseases.

**Figure 2:**
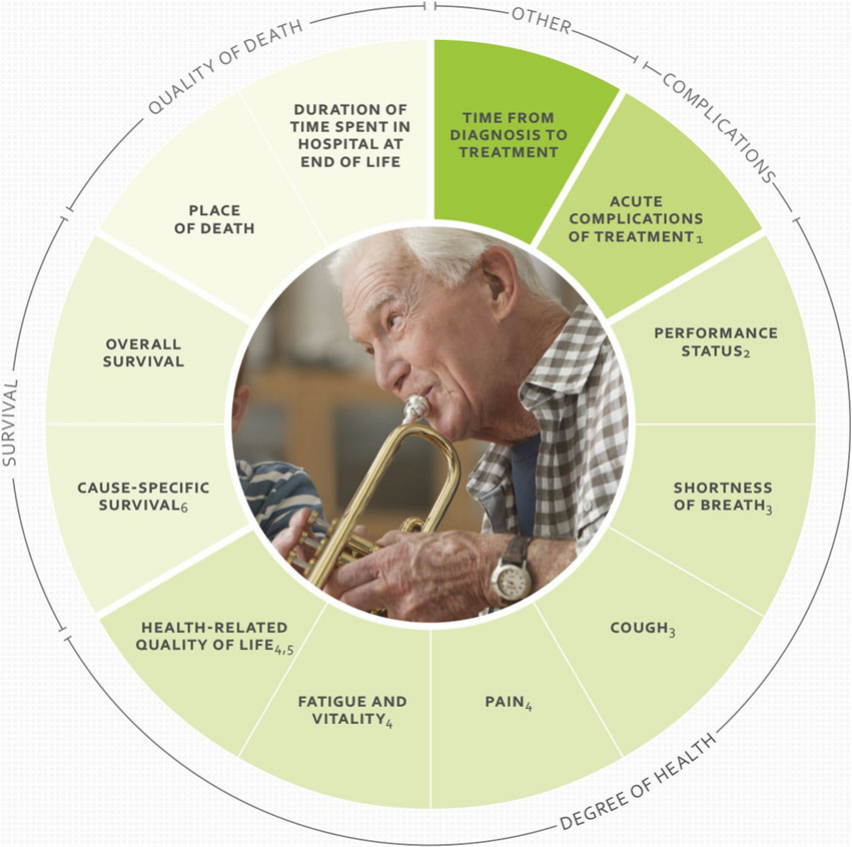
ICHOM standard set for outcome measurement in lung cancer. Source: ICHOM (2017).

Although such multi‐dimensional sets of outcome measures presented in Fig. 2 may be necessary for capturing all relevant aspects of health care for a cancer patient, and thus are relevant for providers of services to these patients, they are not useful as information to support decisions about allocation of resources. If an aggregate outcome should be related to costs, and thus provide a measure of efficiency, there is a need to assess how each dimension is affected and the relative importance of each dimension. Even if such an index could be constructed, it would be difficult to interpret the calculated ratio, and thus it would not give any useful information.

An alternative approach is to define an effectiveness or outcome measure that can be related to costs in a way that it gives relevant information. There are several options for this. If the primary objective is to cure patients, costs could be related to number of patients cured. Alternatives that cure many patients per unit of cost are better that those that cure fewer patients. In practice, economists prefer to calculate and report the inverse, the cost per cured patients, as a measure of cost‐effectiveness.

Mortality can be used as an outcome measure, but the time perspective is important, as in the long‐run we are all destined to die. But cost per additional survivor at 1 or 5 years could be relevant. But such a measure is indifferent to anything that happens before or after the chosen cut‐off point.

Survival is a better outcome, and estimates of cost per life year gained are a relevant measure of effectiveness when the main purpose of the intervention is to prolong life. Whereas outcomes are typically presented in clinical trials as the difference in median survival times, the measure required for economic evaluation is the mean difference (Davies *et al*., 2012). This increases the uncertainty around the estimate of OS gain and thus the estimated cost‐effectiveness ratio.

The issues and problems around estimating cost per life year gained are illustrated in a study of the drug price per life year gained from new cancer drugs introduced in the USA (Howard *et al*., 2015). Cost of drugs is only part of the total treatment costs for the relevant episode of care, which in most cases is the life expectancy for the patient. Calculating cost of drugs based on available prices and assumptions about treatment patterns creates a number of uncertainties. Data on overall median survival are often not available and thus need to be approximated with gains in median progression‐free survival. Gains in mean survival can be modelled, but with additional uncertainty as a result. It is thus not surprising to see a huge variation between observations.

**Figure 3:**
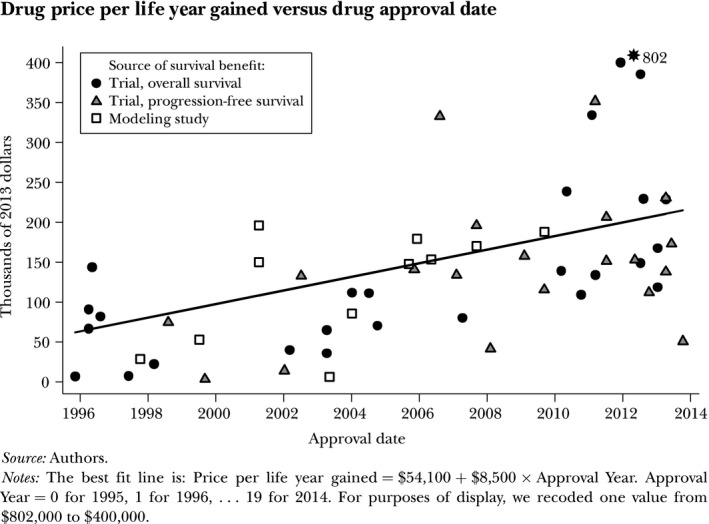
Cost per life year gained from new cancer medicines. Figure reproduced from Howard *et al*. (2015). © American Economic Association; reproduced with permission of the *Journal of Economic Perspectives*.

The drug price per life year gained increased by USD8500 for each year between 1996 and 2013 (Fig. 3): in 1995, patients and their insurers paid USD54 000 for a year of life and in 2013 they paid USD207 000. This may indicate an increase in profitability, but a more probable explanation is a reduction in research productivity, i.e. increasing fixed costs per patient treated to bring a new product to the market. An important factor behind this is the increasing share of new drugs for small patient populations (orhan drugs).

Gain in survival is an important outcome, but increases in survival ignore improvements in quality of life, as well as the fact that prolongation of life can be of different quality. For this reason, health economists and decision makers favour the calculation of a composite measure of survival and quality of life – QALY.

This measure has a similar construction as the DALY, but there are important differences both in the method of calculating the gain in survival and in how survival is adjusted for quality of life. Estimates of QALY lost due to cancer as part of studies of cost‐effectiveness of new treatment alternatives indicate that quality of life plays a larger role than morbidity does in the calculations of DALY lost due to cancer.

**Figure 4:**
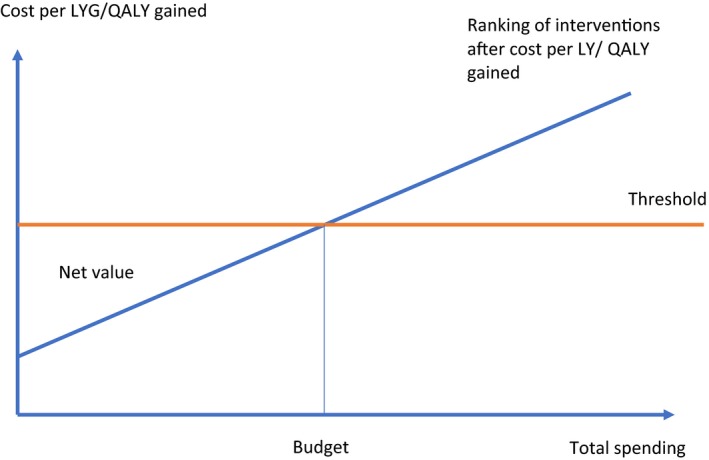
Ranking and selecting new interventions in cancer based on cost per QALY gained.

Increase in the cost/QALY threshold value (shift upwards in red line in Fig. 4) and or improvements in cost‐effectiveness of interventions at the margin (shift downwards of blue line) increase the total spending (budget) on management of cancer. Cost reductions for intra‐marginal programmes, for example as a consequence of price reductions, increase the net value. Spending on interventions with a higher cost/QALY than the threshold, thus reducing the spending on other with a lower cost/QALY than the threshold, reduces the value for patients and the population served.

The methodology behind calculation of QALY is not universally accepted, but it is enough well developed and accepted to be considered a relevant metric for a mission‐oriented translational cancer research policy. This does not mean that we assume that QALY includes all relevant outcomes from investments in cancer research and cancer care.

## 6 Assessment of cost‐effectiveness at adoption of new interventions to the healthcare system

With a new treatment, there are no data available from its use in clincal practice. Cost‐effectiveness must thus be assessed through models simulating the potential effectiveness and costs for the intended patient population(s). As cost‐effectiveness is a comparison with existing (best) practice, it is necessary to have data on how resources for cancer control/management are spent and related to outcome in the specific population. Data on resource use and costs for current standard of care are also important, as new therapies will substitute and complement existing alternatives, and it is the incremental costs and outcomes that define the relevant cost‐effectiveness ratio. When a new alternative is better and less expensive, the decision is easy, and no ratio needs to be calculated. But in most instances, new interventions for cancer control, both preventive and curative, come at an increased net cost to achieve the increased outcome.

**Figure 5:**
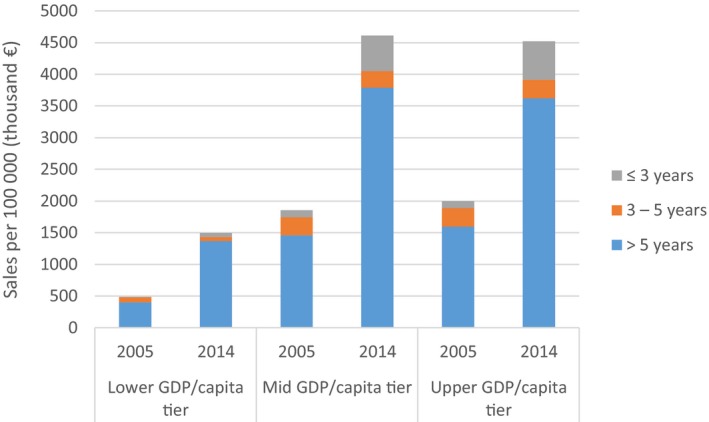
Sales of oncology drugs per 100 000 inhabitants by time since launch by economic status of the country. Source: Jönsson *et al*. (2016b).

Costs should also have a social perspective and should include indirect costs for loss of production as well as direct costs outside the healthcare system regardless of payer. The problems involved in early assessment of cost‐effectiveness of new cancer treatments are well known (Jönsson, 2013, 2015). Oncology presents specific challenges, linked to the need to make health economic assessments very early in development. Cost‐effectiveness assessment requires estimates of gains in mean survival, whereas clinical trials are designed to study differences in progression‐free or overall median survival. This increases the uncertainty in the estimate of survival gain and thus cost‐effectiveness. The development of targeted therapies and personalised cancer medicine increase the complexity of the assessment. Smaller and shorter trials may give safer and faster evidence about which treatment may work for different types of patients, but they will not provide enough information for assessment of outcome and cost‐effectiveness. Assessing a diagnostic and a new treatment together increases the number of alternative intervention strategies, which requires additional studies and data.

The traditional clinical trial approach of using progression‐free survival and cross‐overs has serious shortcomings, producing data that cannot be used to determine outcomes and, so, cost‐effectiveness. A new standard is needed which must be developed in collaboration with clinical researchers and health economists, involving both regulatory and HTA authorities. Decisions about payment and adoption must be made under conditions of great uncertainty in predictions about outcome, but providing relevant information before adoption is key for creating value for patients in the translational research process.

Looking to the next decade, the focus must be on economics being integrated into real‐world studies, as well as better integrating it into the clinical studies and trials. Twenty years ago it was acceptable to conduct clinical trials in cancer that did not look at any biological endpoint; today nearly every trial is biologically driven and/or has a biological add‐on study. Yet health economics is not normally integrated or is often done *post hoc*.

## 7 Follow‐up studies of cost‐effectiveness of new interventions when used in the healthcare system

While the design of clinical trials and collection of relevant data informs early decisions about payment and adoption, uncertainty about consequences in clinical practice is an issue that must be addressed. First, careful planning and performance of such studies may reduce the time and costs in the translational research process. Secondly, such studies may not only confirm or reject early predictions but also contribute to increased value of the new treatment and prevention options. Creating optimal value of an intervention is seldom a question that can be answered with a yes or no but is more about which patients to treat and making adjustments in treatment regimens as new evidence and alternatives develops. For prevention programmes, it is about details of which populations to target, and which screening intervals and cut‐off values should be used for further interventions. As value in actual use determines the returns to investments in research and development, collaboration with the healthcare system becomes an integral part of the translational research process.

Traditionally, the healthcare system has not been involved in research: ‘It seems taken for granted that the technology of medicine simply exists, take it or leave it, and the only major technologic problem which policy‐makers are interested in is how to deliver today's kind of health care, with equity, to all the people’ (Thomas, 1971). Today it is obvious that decisions about introduction and use of new options for prevention and treatment, based on research and development, are a major factor behind improvements in outcomes and cost‐effectiveness, and thus a key factor for delivering value‐based health care. At the same time, those decisions will determine the returns to investments and thus provide feedback and incentives for further development.

However, the healthcare system is not yet prepared for this new role. Although data on use of resources and actual outcomes from specific interventions are rapidly evolving, and methods for comparative effectiveness analysis are being presented, the actual use of such studies is still in an early stage (Jönsson *et al*., 2014; Luce *et al*., 2010). There are many reasons for this. One is a lack of interest, requirements and availability of funding for such studies. A study by Davis *et al*. (2017) reported that of the cancer drugs approved by the European Medicines Agency between 2009 and 2013, 57% (39/68) had no supporting evidence of better survival or quality of life when they entered the market. After a median of 5.9 years on the market, just six of these 39 (15%) agents had been shown to improve survival or quality of life (Davis *et al*., 2017).

When follow‐up studies are required, the data and methodology for providing conclusive information are lacking. Franken *et al*. (2014) investigated whether policymaker uncertainty regarding cost‐effectiveness was reduced by using data on real‐world usage of bortezomib in the Netherlands. The authors concluded that much of the uncertainty regarding the real‐world cost‐effectiveness of bortezomib remained after outcomes research and that policymakers should carefully consider whether some sort of risk‐sharing agreement would be better at reducing the uncertainty. Another lesson learned from that study is that post‐market access outcomes research requires sufficient data, which in turn requires a clear treatment strategy for a given drug. One problem the authors encountered was that a comparison between patients receiving bortezomib and patients not receiving bortezomib was impossible because of the extensive treatment variation, e.g. regarding line of treatment, missing data and the lack of a general treatment strategy. These findings highlight the need for sufficient documentation of treatment for each individual patient and the need for clinical practice guidelines to be followed in order to facilitate research on the real‐world cost‐effectiveness of a drug. It also highlights the potential difficulties of conducting cost‐effectiveness studies using real‐world data without a pre‐determined study design.

## 8 Value, price and payment for new cancer medicines

Value is what you get and price is what you pay. Pricing determines how value is distributed and also gives incentives for innovation through actual or potential impact on profits. Economists agree that the magnitude of actual and expected profits impact the magnitude of private for‐profit research in the life science industries. For cancer research, the main payers are the private and public healthcare systems in different countries. The total market for cancer medicines was USD133 billion in 2017 and is growing at a rate of 10–15% per year. In the EU, spending on cancer medicines was about €20 billion in 2014, which accounted for about a quarter of the total spending on cancer (Jönsson *et al*., 2016a,b). The willingness by healthcare systems to devote an increasing share of resources to cancer medicines is driving private investments in research and development.

The ability of profits to guide firms toward the most socially valuable kinds of R&D is highly dependent on two factors: (1) how well‐informed healthcare systems, doctors and patients are about the value of existing drug and new drugs and (2) how strong incentives are for payers, doctors and patients to consider price when choosing between cancer medicines and other therapeutic options. Well‐informed decisions about payment for new cancer medicines are crucial for the outcome, efficiency and value of these investments. Value‐based pricing and reimbursement and estimates of cost‐effectiveness are used as an instrument to guide payment decisions.

A problem for value‐based pricing is that the value of a new medicine or other medical technology is not known when it comes to the market. There is great uncertainty at that time, which makes it difficult to assign a value‐based price. However, payment systems can be designed for management of uncertainty, in order to create both access for patients and optimal use, as well as proper incentives for innovation by rewarding those who develop drugs with validated outcomes and high value for patients.

Traditionally, price is fixed per unit sold of the medicine. As new medicines are introduced with ever higher investments before the first unit is sold, the difference between price and marginal cost are becoming greater. The role of pricing is mainly to recover sunk costs, and as a consequence there is a growing inefficiency (‘excess burden’) due to patients not having access to new medicines when available even if they are willing to pay the additional marginal costs for access. Financing investments in R&D by pricing the medicine above marginal cost means that decisions about the use of new medicines will be inefficient.

This is a particular problem in Europe, where there is a common market but not a common healthcare system. As a consequence, new medicine is launched at a similar price, and there is very little use of these medicines by countries with lower incomes per capita, compared with the richer countries.

Differences in ability to pay determine the variations in use, also of medicines with established and value, but this is not the only factor behind the variations. The vast heterogeneity across EU around policies for supply and demand, has a side effect on how the private and public sectors invest and work in individual countries. Establishment of a common system for assessment, introduction and follow up of new cancer medicines is needed for efficient use and equal access. A new system for payment for cancer medicines, based on the principle of separating payments for sunk costs for R&D and payments for use of the medicine, is also needed for efficient use and equal access to valuable new medicines. Such a system is already developing through higher rebates on list prices, price volume agreements, and other forms of market access agreements, but needs to be more transparent and evidence‐based.

## 9 Discussion and Conclusions

We have for a very long time observed great variation in cancer care between and within countries. This was expected in a situation when there was little evidence of what worked, and where development of local practices was both natural and accepted. As the difference in costs for the various alternatives was rather small, and the medical decision‐making complex for severely ill patients, variations in clinical practice within and between countries were accepted without many questions.

But when there is evidence about what works and not works for different patients, and, in addition, costs related to these different options are very high, the tolerance for such variations diminishes (shifts towards ‘precision medicine’). But, at the same time, the complexity of new treatment options, often in combination and/or sequence, makes decision making more difficult.

There is a cry for more data to inform those decisions. But data are not enough; there is a need for methods for analysis and mechanisms that make healthcare systems responsive to results. There is a great deal of evidence that allocation resources are based more on local interpretation of clinical studies and economic incentives for different actors in the healthcare system, than on systematic evidence about clinical outcomes and cost‐effectiveness.

This is of great importance for translational research. New technologies must be implemented correctly to create optimal value for patients. Over‐use of ineffective technologies takes away opportunities for new methods to be introduced. Traditional HTA, based on systematic review of a large number of clinical trials, has an important role to help inform about potential dis‐investments, but new data and decision‐making criteria and processes are needed for decision making on new options for cancer management. The focus has been on new cancer medicines, but equal interest must be devoted to how non‐pharmaceutical technologies are impacting direct costs and also how they alter the systems, making them both more and less efficient.

The most important objective for translational cancer medicines is to produce new cost‐effective alternatives for cancer prevention and treatment. If this is achieved, there will also be support for increasing resources for this. However, it is also important that the costs in the development process are controlled. There is evidence of a reduced productivity over time, making new medicines that come to the market more and more expensive. We need more evidence about this development, and identification of policies that can reverse this trend. With new information technology, it should be possible to reduce costs for clinical trials through collaboration between the private developers and the healthcare system. Many of the data needed to inform decisions about resource allocation in health care, for example about patient outcome in relation to defined interventions, can be used in the translational research process.

Health economics provides a link between decision making in the healthcare system which determines the value and cost‐effectiveness of different options for prevention and treatment. This is important for informing decisions about investments in cancer research for the benefit of patients. New options for cancer management can no longer be seen as an exogenous factor that is dealt with on an *ad hoc* basis. Development of new options for prevention and treatment is now an integrated part of health care, with consequences for both outcomes and costs. Health economics is an integrated aspect of all phases of mission‐oriented translational research, necessary for creating the link between early decision making and adaption of new technologies, follow up of outcomes in clinical practice, and early decision making on the design of clinical studies and outcomes used.

## Conflict of interest

The authors declare no conflict of interest.
